# Effectiveness and safety of different academic schools of traditional Chinese medicine in the treatment of obesity: A protocol for systematic review and meta-analysis

**DOI:** 10.1097/MD.0000000000031960

**Published:** 2022-12-09

**Authors:** Xiaochao Gang, Tianjiao Gao, Yiran Han, Yuxing Tai, Chongwen Zhong, Shaotao Chen, Ying Gao, Lijie Li, Zhenxiang Xiao, Dilnur Barat, Mingjun Liu

**Affiliations:** a Changchun University of Chinese Medicine, Changchun, China; b Changchun University, Changchun, China; c Acupuncture and Massage Center of the Third Affiliated Clinical Hospital of Changchun University of Chinese Medicine, Changchun, China; d Third Affiliated Clinical Hospital to Changchun University of Chinese Medicine, Changchun, China.

**Keywords:** academic schools of traditional Chinese medicine, meta-analysis, obesity, protocol

## Abstract

Obesity is a global epidemic. Since 1975, the global obesity rate has almost tripled. Although many systematic reviews and clinical trials have shown that traditional Chinese medicine (TCM) can effectively treat obesity, the effectiveness and safety of different academic schools of TCM in treating obesity have not been systematically evaluated.

**Methods:**

The retrieval language of this study was Chinese and English. From the date of creation of the following data to June 2023, the data of Medline, PubMed, Embase, Cochrane Science Network, China Biomedical Literature Database, Central Controlled Trial Registration Center, and China Science Journal Database were retrieved, respectively. This study included clinical randomized controlled trials related to the treatment of obesity by different academic schools of TCM. The main outcome measures were body mass index, waist circumference, hip circumference, waist hip ratio, body fat content, fasting blood glucose, glycosylated hemoglobin, and blood lipid level. In addition, we manually searched other resources, including reference lists of identified publications, conference articles, and gray literature.

**Results:**

This study will provide a more diverse choice of treatment options.

**Conclusion:**

The purpose of this study is to summarize and evaluate the effectiveness and safety of different academic schools of TCM in improving and treating obese patients from clinical trials, so as to provide more options for obesity treatment.

## 1. Introduction

Obesity is caused by the fact that the intake of calories is greater than the consumption, and the excess calories are converted into a large amount of fat, so that the weight exceeds the normal standard. Among them, simple obesity belongs to non-pathological obesity, excluding other diseases.^[1]^ In recent years, obesity has become a common disease all over the world, and it is easy to cause a variety of acute and chronic diseases that endanger human health, such as hypertension, hyperlipidemia, type 2 diabetes, insulin resistance, coronary heart disease, etc.^[2,3]^ Therefore, it is very necessary to treat obesity. At present, it is urgent to find a safe and effective treatment. From 1984 to 2003, there were about 40 million obese patients in China, and there were more obese patients in northern China than in southern China, which was related to the climate, lifestyle, eating habits and genetic factors of residents in northern China.^[4–8]^ Obesity is not only found in adults, but also in more and more children and adolescents. By 2019, the number of obese people under the age of 17 in China has increased to about 550,000, of which 88% are overweight teenagers and 12% are overweight children. At present, there are many methods to treat obesity, such as gastric restriction, gastric banding, gastric Roux-en-Y bypass, lifestyle intervention, drug intervention, etc. Traditional Chinese medicine (TCM) is gradually playing a certain role in the treatment of obesity.^[9,10]^

Different academic schools of TCM use different methods to treat and improve the symptoms of obese patients, which have different characteristics and clinical effects, including TCM, acupuncture, massage and so on. Animal experimental research shows that massage therapy of TCM can affect fat metabolism by improving the expression level of PPAR-Y in subcutaneous fat cells, thus treating simple obesity. It is believed that acupuncture and catgut embedding therapy can enhance the excitability of VMH in the satiety center of ventromedial hypothalamic nucleus, suppress appetite and improve the symptoms of obese patients.^[11]^ Although a number of systematic reviews and clinical trials have suggested that TCM can effectively treat obesity, the effectiveness and safety of different academic schools of TCM in treating this disease have not been systematically reviewed. This meta-analysis aims to summarize and evaluate the clinical efficacy and safety of different academic schools of Chinese medicine in treating obesity, and provide more treatment options for clinicians and patients.

## 2. Methods and analysis

This systematic review has been registered in the PROSPERO network (No. CRD42022367880). All steps of this systematic review will be performed according to the Cochrane Handbook (5.2.0).^[12]^

### 2.1. Inclusion criteria

#### 2.1.1. Types of studies.

The literature adopted in this study includes blind research types, all of which belong to random clinical research test types. In order to ensure the accuracy of the results, meta-analysis, animal experiment, literature review and case literature were excluded.

#### 2.1.2. Types of participants.

The screening of participants needs to exclude obesity caused by diseases, taking drugs, pregnancy and other factors, and all participants need to be diagnosed as obesity. However, there are no excessive restrictions on gender, age, nationality and other factors.

#### 2.1.3. Types of interventions and comparisons.

The treatment and intervention methods used in the experimental group included different academic schools of Chinese medicine, such as acupuncture, massage and oral Chinese medicine. The intervention methods of the control group can include oral western medicine, placebo, etc.

#### 2.1.4. Types of outcome measures.

Results The main measurements will be body mass index, ideal weight and obesity, waist circumference, waist-hip ratio and body fat content. In addition, fasting blood glucose, glycosylated hemoglobin and blood lipid levels will also be included in the assessment.

### 2.2. Search strategy

Search PubMed, Embase database, ScienceNet, Cochrane Central Registry of Controlled Trials, China National Library Knowledge Infrastructure, China Biomedical CD-ROM, China Clinical Trials Registry database, China Scientific Journal database and Wanfang database, from the date of establishment of the above databases to the end of December 2022, including other relevant research materials. Search keywords include “Simple obesity,” “obesity,” “academic schools of Traditional Chinese Medicine,” “traditional Chinese medicine,” “Controlled clinical trial” and so on. Table 1 shows the search strategy in databases.

**Table 1:** Search strategy used in Pubmed database.

Number	Search terms
#1	Obesity (All files)
#2	Simple obesity (All files)
#3	#1 OR #2
#4	Academic Schools of Traditional Chinese Medicine (All files)
#5	Traditional Chinese Medicine
#6	TCM (All files)
#7	#4 OR #5 OR #6
#8	Randomized controlled trial (All files)
#9	Controlled clinical trial (All files)
#10	Randomly (All files)
#11	Randomized (All files)
#12	Placebo (All files)
#13	Double-blind method (All files)
#14	Single blind method (All files)
#15	Trials (All files)
#16	#8 OR #9-15
#17	#3 AND #7AND #16

### 2.3. Study screening and data extraction

**Figure 1.**
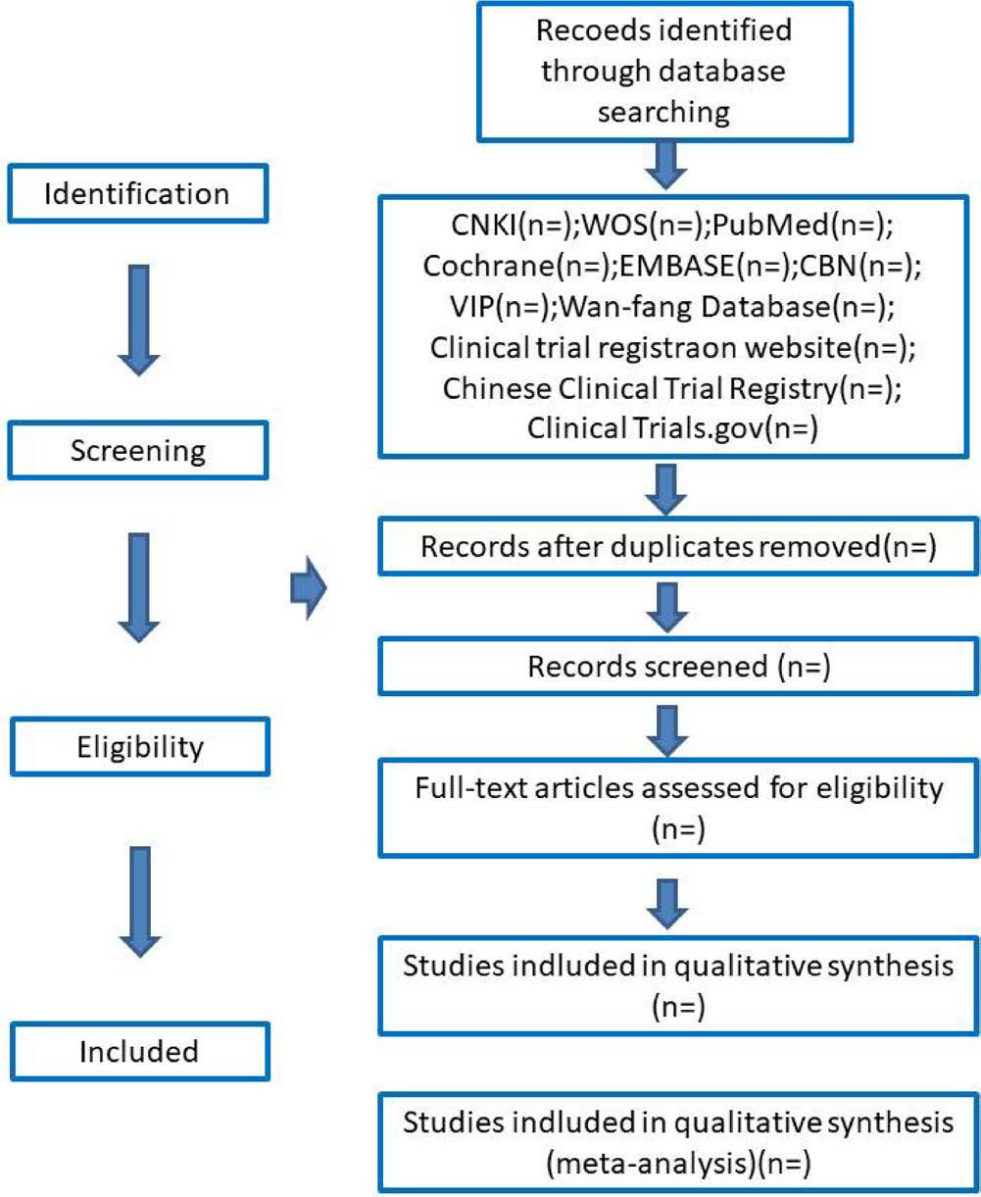
Flow diagram of study selection process.

After the preliminary data is extracted, it is screened to eliminate duplicate content. Then, 2 reviewers independently screen the titles and abstracts according to the qualification criteria to construct an extraction list. For the data with incomplete information, we should contact the providers of these data to supplement and improve the data. When different opinions are found in the retrieved data, a third person will judge and finally make a conclusion as an expert. The process of identifying and selecting documents is shown in Figure 1.

### 2.4. Statistical analysis

The flow chart is created by PRISMA scale and Review Managerversion 5.3. The meta-analysis software was Revman 5.3 and Stata 16. Weighted mean differences were calculated with 95% confidence intervals for continuous variables. The *Q* test and *I*^2^ test were used to assess the heterogeneity of the data. When heterogeneity was not significant (*P* ≥ .10 or *I*^2^ < 50%), a fixed effects model was used to analyze the pooled effect, and when heterogeneity was statistically significant (*I*^2^ ≥ 50% or *P* < .10), a random effects model was used Reasons for heterogeneity were explored by subgroup analysis.^[13]^ During the research process, researchers explored the root causes of heterogeneity through sensitivity analysis. In case of serious heterogeneity, check whether the data are correct again, such as whether there are errors in the analysis unit, or properly exclude a small number of related studies on possible sources of heterogeneity, so as to effectively reduce heterogeneity. Evaluation of publication bias. If more than 10 articles are included in the meta-analysis, the researcher will draw and analyze the funnel chart by using Revman 5.3, and observe whether the funnel chart is symmetrical, so as to judge whether the evaluation results are biased. The research results are illustrated by forest map.

### 2.5. Sensitivity analysis and grading of evidence quality

By analyzing the quality of research methods, sample size and other contents, the sensitive contents in the research review process are judged and decided. The quality of the evidence involved in the study is divided into 4 grades: high, medium, low and polar.

## 3. Discussion

Obesity is a chronic and recurrent metabolic disease. In recent years, the prevalence of obesity in China is on the rise. There are many causes of obesity, including genetic factors, such as gene variation and leptin resistance, gene variation of adrenoceptor, abnormal energy metabolism, abnormal appetite regulation network, etc. One of the important causes of obesity is the change of human adipocytes. Adipocytes are not only energy reservoirs, but also secrete soluble biological signals such as important hormones, cytokines, vasoactive peptides that lead to obesity. When these biological signals are abnormally transmitted, they may lead to abnormal interactions that mediate brain fat axis, pancreatic islet fat axis, and so on, leading to energy metabolism Transduction of cell signal and gene expression is more conducive to the increase of fat accumulation in the body, thus leading to obesity.^[14,15]^ Research shows that bad dietary habits, such as intake of fat and imbalance of trace elements, may lead to obesity. As a complementary and alternative therapy, TCM treatment of obesity has been proved to have a relatively good improvement and therapeutic effect through a large number of clinical trials. Therefore, this study focuses on collecting and sorting out the clinical treatment plans for obesity in different schools of TCM, aiming to summarize and evaluate their effectiveness and safety, and provide more options for clinicians and patients to treat obesity.

## Author contributions

**Conceptualization:** Xiaochao Gang, Yiran Han.

**Data curation:** Yiran Han,Tianjiao Gao,Ying Gao.

**Formal analysis:** Shaotao Chen, Yuxing Tai.

**Methodology:** Chongwen Zhong, Shaotao Chen.

**Software:** Zhenxiang Xiao, Dilnur Barat, Lijie Li.

**Supervision:** Mingjun Liu.

**Writing – original draft:** Xiaochao Gang, Tianjiao Gao.

**Writing – review & editing:** Mingjun Liu.
